# How to use the world's scarce selenium resources efficiently to increase the selenium concentration in food

**DOI:** 10.1080/08910600701698986

**Published:** 2007-11-27

**Authors:** Anna Haug, Robin D. Graham, Olav A. Christophersen, Graham H. Lyons

**Affiliations:** 1 Norwegian University of Life Sciences, Ås, Norway; 2 University of Adelaide, Waite Campus, Australia; 3 Oslo, Norway

**Keywords:** selenium, essential nutrient, deficiency, fertilizer, food processing

## Abstract

The world's rare selenium resources need to be managed carefully. Selenium is extracted as a by-product of copper mining and there are no deposits that can be mined for selenium alone. Selenium has unique properties as a semi-conductor, making it of special value to industry, but it is also an essential nutrient for humans and animals and may promote plant growth and quality. Selenium deficiency is regarded as a major health problem for 0.5 to 1 billion people worldwide, while an even larger number may consume less selenium than required for optimal protection against cancer, cardiovascular diseases and severe infectious diseases including HIV disease. Efficient recycling of selenium is difficult. Selenium is added in some commercial fertilizers, but only a small proportion is taken up by plants and much of the remainder is lost for future utilization. Large biofortification programmes with selenium added to commercial fertilizers may therefore be a fortification method that is too wasteful to be applied to large areas of our planet. Direct addition of selenium compounds to food (process fortification) can be undertaken by the food industry. If selenomethionine is added directly to food, however, oxidation due to heat processing needs to be avoided. New ways to biofortify food products are needed, and it is generally observed that there is less wastage if selenium is added late in the production chain rather than early. On these bases we have proposed adding selenium-enriched, sprouted cereal grain during food processing as an efficient way to introduce this nutrient into deficient diets. Selenium is a non-renewable resource. There is now an enormous wastage of selenium associated with large-scale mining and industrial processing. We recommend that this must be changed and that much of the selenium that is extracted should be stockpiled for use as a nutrient by future generations.

## Introduction

Selenium (Se) is a rare element on our planet, with the average concentration in igneous bedrock being only 0.05 mg/kg (1), which is less than for any other nutrient element. The world's Se resource is limited because there are no ores from which Se can be mined as a primary product (2).

The Se level in food depends on several geological, geochemical and climatic factors (1,3–12). Selenium enters the food chain through plants, and the Se concentration of plants varies according to available soil Se concentration, its bioavailability for uptake into plant roots (which depends heavily on redox equilibria in the soil, but also on several other factors) and species of plants. A worldwide atlas illustrates the variability of Se distribution globally (13).

Selenium is critical to the health of living organisms. It has been postulated that the vast majority of the world's population has suboptimal Se intakes, and hence is at increased risk of several diseases such as cancer, heart disease, viral diseases and other conditions that involve increased levels of oxidative stress (14). There are several disease conditions (e.g. diabetes, several infectious diseases and possibly asthma) where the disease itself and the enhanced oxidative stress may be caused primarily by factors other than Se deficiency *per se*, but where good Se status in combination with an adequate intake of other antioxidative nutrients may help cells and tissues better to cope with harmful oxidative stress caused, for instance, by some toxic heavy metal (15,16) or other environmental pollutants, by hyperglycaemia (17), or by the immune system's reaction to infection (18). Efforts to increase Se concentration in the diet are urgent for both current and future generations.

Selenium is also a strategic element for high technology applications, and industry has great demand for the mineral. Approaches to improve production and recycling of Se or limitations in use may be necessary to satisfy demands for both health and industry. A discussion of some ways to improve efficiency of use of Se in agriculture and food is warranted.

The object of this commentary article is to focus on some of the ways to increase Se concentration in diets, and to discuss briefly the utilization of the planet's scarce Se resources for human health and well-being, as well as animal health and plant food quality.

## Selenium concentration in bedrock and soils

Selenium is unevenly distributed over the surface of the Earth, ranging from near zero to 1250 mg/kg. In igneous and metamorphic rocks and in sulphide ores, Se is found almost exclusively (as selenide ions) in sulphide minerals, including iron, nickel, copper, zinc and lead sulphides. However, sedimentary rocks may contain oxidized Se species such as selenite or selenate, which are also the most common Se species in soils (in addition to organic Se compounds, especially in living organic matter). The average Se concentration is much higher in sedimentary rocks, especially shales and coal, than in igneous rocks (1). This can be explained as a consequence of volatile Se transfer to the atmosphere and hydrosphere during volcanic processes.

About 30 years ago, by studying Norwegian soil samples Norwegian researchers found that there was a high positive correlation between soil concentrations of Se and iodine (5,6). It had been known for a long time that the oceans – via evaporation, atmospheric transport and deposition from rainwater or snow – are a major source of iodine in soils, with the supply rate being much higher in humid coastal areas than inland (19). It was then demonstrated, however, that supply from the sea via rain and snow was as important for Se as it earlier had been shown to be for iodine, and the Se concentration was much higher near the coast than inland (5,6,20). In addition it was shown that sulphuric acid-rich polluted rain was an important source of Se (20). The mechanism for transfer of Se from the sea to the atmosphere was not known at that time, but later it was shown that it takes place by evaporation of dimethylselenide and other volatile Se compounds (21,22), similarly as iodine is transported from seawater into the atmosphere in the form of methyliodide (23,24).

Areas with unusually high soil Se concentrations include parts of Wyoming and North and South Dakota in the USA (25), Enshi County in China and parts of Ireland, Colombia and Venezuela (14). In some of these regions, the principal cause of high soil Se concentrations is high local bedrock concentrations. But Se in soil comes not only from the local bedrock, but also from atmospheric deposition, and in the coastal climate of Ireland it is more likely that high soil Se is caused by accumulation of Se deposited from rainwater over a very long period of time. In a few areas high, toxic levels originate from industrial activity in the area, but more often they occur naturally (13), with selenosis most commonly occurring when domestic animals eat Se accumulator plants.

In many parts of Europe, soil Se concentrations are relatively high because of high deposition either naturally from the sea (e.g. Ireland, England, Scotland and the Netherlands) or from polluted rains (e.g. Germany, the Czech Republic, Slovakia and Poland). In many districts in Western, Central and Southern Europe, local bedrock Se concentrations would also be expected to be relatively high, due to the abundance of surface outcrops of marine sediments often of Mesozoic or Cainozoic age. From a geochemical point of view, there is therefore little reason to believe that soils in western and central Europe are on average much more Se-depleted than soils in North America.

Finland (until 1984, before Se was added to fertilizers), central Serbia, a belt from north-east to south-central China (26) and parts of Congo (27) are notably Se-deficient. In Finland and Serbia, as in other parts of Europe, the most important factor is probably poor uptake in the plants. But in central China and central/eastern Congo, the main problem must be the low concentration of Se in the soil. Large areas of Africa are likely to be Se-deficient, but further mapping is required. Australia has both high-and low-Se soils and large areas have not been mapped. It can be concluded that large areas of the world have suboptimal levels of Se in their food systems (14,28).

## Bioavailability of soil Se for uptake in plants

Agricultural crops can be Se-deficient either because of low Se concentration in the soil or because of poor availability of soil Se for uptake into the plant roots (or a combination of both factors). Poor uptake seems often to be the principal cause of Se deficiency in plants grown on cultivated lands in industrial countries (where there are large regions with medium to high soil Se concentrations, but still very little Se in the food and forage crops grown on cultivated lands), while Se deficiency in the soil is the most common cause of severe Se deficiency in poor countries, such as in parts of Sub-Saharan Africa. As a consequence of the expansion of modern forms of agriculture in former poor countries, especially in Asia and Latin America, it may be possible that many of these countries could now be facing a similar problem to that in Europe, with the bioavailability of soil Se on cultivated lands now often being lower than before. This problem is likely to become even more prevalent in the future, as a consequence of population and economic growth.

Selenium form is important for bioavailability: selenate is less strongly adsorbed to minerals in the soil and more readily taken up by plants than selenite. Many factors such as dry climate, low organic matter concentration in the soil, high temperature, high pH and no water-logging may give a high ratio between selenate and selenite in the soil. Selenite, however, is the dominant form of inorganic Se in soils with high concentrations of organic matter, as in the Nordic countries (because of low soil temperatures causing much slower degradation of soil organic matter than in tropical countries), and most likely also in waterlogged soils (during rice cultivation).

In New Zealand, Se deficiency in the plants can be explained by a combination of poor uptake and low Se concentrations in volcanic bedrocks. Selenite ions are strongly adsorbed not only to ferric oxide/hydroxide minerals, but also to allophane formed by weathering of volcanic glass (11). Selenium status of plants and livestock in New Zealand declined after manufacturers removed arsenic from superphosphate fertilizers. The process that was used for removal of arsenic took away Se as well, and the colour of the fertilizer changed from pink (because of finely disseminated elemental Se) to white or grey. It took only a short time before sheep started to develop symptoms later known to be caused by Se deficiency and to die in such large numbers that in some places they had to be buried by the thousands in large mass graves by bulldozers (Douglas V. Frost, personal communication).

One hypothesis that may explain why low Se is sometimes associated with fertilizer application may be co-precipitation of selenite ions with phosphate. This renders much of the Se unavailable for uptake by plants (29–31). When new phosphate fertilizers are added to the soil, and new precipitation of phosphate minerals takes place, Se remains fixed in the precipitate and unavailable for uptake. Conversely, phosphate may also lead to desorption of selenite ions bound to minerals in the soil, as phosphate is bound more strongly to trivalent iron and aluminium than is selenite (31,32). In the USA and Canada, by contrast, there are large areas of prairie land where the soil has been so fertile that application of large amounts of phosphate fertilizers has been unnecessary. The high concentrations of Se found in cereals grown on these lands can therefore be explained by a combination of high natural Se concentration in the soil and excellent bioavailability of soil Se for uptake by the plant roots, due to lack of commercial P fertilizer application.

Selenium concentrations in plants may, furthermore, be reduced following application of sulphate as a result of competition between sulphate and selenate for transporters in plant roots (33). Selenium and sulphur (S) compete with each other in the biochemical pathways, leading to synthesis of selenomethionine (Se-met) and methionine in plant cells. The concentration of Se-met in plant seeds must therefore be expected to depend strongly on the ratio of Se uptake to S uptake in the roots. In the more humid parts of Sub-Saharan Africa, there are large areas where the human diet is deficient in S amino acids (34), which is in part explained by a low total intake of dietary protein (35), but also may happen because of S deficiency in the soil (36–40). If one tries to correct the problem of S amino acid deficiency in the human diet by fertilization with Se-poor sulphur (in the form of gypsum or elemental sulphur to avoid too rapid loss of the S by vertical leaching processes), it may be theoretically expected that this could lead to a reduction of the rate of synthesis of Se-met in the plants. This does not mean that one should not try to correct a problem of S deficiency in the soil. But it may be important to use some other method to enhance the dietary intake of Se (e.g. adding Se to animal feed and thus enhancing the human Se intake through livestock products) – to compensate for the effect of fertilization with S on the Se concentration both in locally produced plant products and in animals eating Se-deficient fodder.

An important unresolved question concerns the mechanism of selenite uptake into mycorrhiza and plant roots, whether there is a specific membrane transporter for the selenite ions, or if selenite and some other more abundant anion, such as phosphate, may share a common membrane transporter. If the latter should be the case, it must be expected that phosphate will function as a competitive inhibitor of selenite ion transport into the plant roots, even in the absence of interactions between the two elements caused by binding (by adsorption or coprecipitation) to minerals in the soil. The same question could also be raised regarding the mechanism of active transport of selenite ions into other types of organism, e.g. into various groups of planktonic algae or into mammalian cells such as erythrocytes.

The low Se concentrations found in plants grown on agricultural lands in Europe (in most parts of northern, western and central Europe with the exception of Finland) cannot be explained simply as a consequence of Se-depleted soils. This may be illustrated by the situation along the Norwegian coast, where topsoil (uppermost 10 cm in non-cultivated lands) Se concentrations are so high in some regions as a result of high deposition rates from the atmosphere, both naturally from the sea and from acid rain (20), that they approach levels found in seleniferous areas in USA (with Se poisoning occurring among livestock because of ingestion of Se accumulator plants) (9). But the Se concentration found in plants grown on cultivated land along the Norwegian coast is still very low, even though it may locally be much higher in parts of the forest vegetation, as for example in pine needles eaten by capercailzie (*Tetrao urogallus*, also called wood-grouse, which is a large game bird found in conifer and mixed forests in Scandinavia) (41). Higher Se concentrations than commonly found in plants growing on cultivated land (42) before 1984, when Se fertilization was started, have also been found in trees and other forest vegetation growing on relatively Se-rich soils (because of Se-rich bedrocks) in Finland (43).

## Selenium production

Most of the world's Se is produced in the USA, Japan and Canada, as a by-product from copper mining, with smaller quantities coming from China, Australia and other countries with a copper refining industry. World production has increased considerably since the element began to be used commercially in the early 1900s. The global production in 1910 was about 5000 kg and has increased to an estimated 2300 tonnes per annum (13).

## Consumption of selenium in agriculture, as dietary supplement and by industry

Selenium is an essential trace element for humans and all animals, as well as being needed in many diverse ways in industry. The use of Se in biology and agriculture added to chemical fertilizers, animal feeds, veterinary preparations and as a human diet supplement accounts for only about 5% of total demand (2) but can be expected to increase markedly. Industrial demands for Se account for most of the production, including a wide variety of industrial applications such as electrical, pigment, glass, metallurgical and others being 30%, 10%, 35%, 10% and 10% of demand, respectively.

Selenium has unique electrical properties that make it of special value to industry. Its electrical conductivity, which is low in the dark, is increased several hundredfold on exposure to light. Selenium is a semiconductor, having asymmetrical conductivity that allows it to conduct an electrical current more easily in one direction than another (25), making it important in several electrical devices and also for reducing solar heat transmission. Selenium is also used in glass colour production, production of pigments and in photoreceptors (2).

## Reuse of selenium, and stockpiling for future generations

The use of Se as a minor component in so many products makes it difficult (or impossible) to be recycled efficiently, and only about an estimated 15% of refined Se comes from secondary sources (2). Selenium is economically recoverable from industrial scrap and chemical process residues, and for example, worn out and damaged photoreceptor drums are recycled. Reuse of metals is common for several elements, including copper. If copper is easier to recycle than Se and the percentage of reuse of copper is higher than for Se, we could expect that the relative demand for Se will be higher than for copper in the future, due to the fact that Se is mainly a by-product of copper refining, and that there are no deposits that can be mined commercially for Se alone. Selenium shortage may therefore be a result. At present, Se is fairly inexpensive, the current price of sodium selenate in Australia is in the range of A$120 to $160 per kg (early 2007), a relatively modest price that may not encourage efficient utilization.

There is now an enormous wastage of Se associated with large-scale mining and industrial processing (e.g. nickel and coal) when the Se content of sulphide ores and of coal is not recovered. Hopefully in the near future this will be changed, and we recommend that most of the Se that is extracted should be stockpiled for use as a nutrient by future generations.

## Selenium in human nutrition

### Selenium intake

There has been a downward trend in Se intakes in many European countries in recent years (14,44,45). The reason for this is not completely understood, but substitution of North American wheat imports grown on high-Se soils by wheat from within Europe grown on soils with low Se concentration or much poorer Se bioavailability for uptake in the plants (as compared with the high-Se areas in North America) is widely accepted to be a primary reason (45).

Selenium intake data for a number of countries show that the recommended daily intakes are not achieved in the majority of European countries together with parts of China (46). Human blood concentrations of Se (plasma, serum or whole blood) of healthy adults from 69 countries also indicate that nutritional Se deficiency is highly prevalent in 21 countries, and moderately prevalent in 16 countries (using the modest reference value of 70 μg Se/l in serum or plasma) (14). It is evident that many people do not consume enough Se to support maximum expression of selenoenzymes. It has been suggested that Se concentration is too low in food produced in most of Europe, parts of Africa, Asia, and New Zealand. Combs (14) estimated the number of Se-deficient people in the world to be in the range of 500–1000 million.

Concerns about the toxicity of Se have been raised, and inorganic forms of Se are accepted to be more acutely toxic than organic forms. However, a number of human studies show that up to 800 mg Se/day administered as Se-yeast gave no symptoms of toxicity (46), and about 400 μg Se/day is considered a safe upper limit (47). One report of high dose Se supplementation showed that intakes up to 3200 μg Se/day gave no obvious Se-related serious toxicities in men (48). Therefore it appears that Se is not as toxic as is often believed (47). However, there are anecdotal reports of paradoxical intolerance to Se in patients suffering from mercury poisoning, even at very modest dosage levels. A transient decline in platelet glutathione peroxidase was also found during resupplementation with Se in test persons who had participated in a trial of experimental Se depletion and repletion (49). One possible explanation could be auto-inhibition of Se-dependent enzymes because of selenite ions reacting with selenol groups in the enzymes (with formation of Se-Se covalent bonds causing inactivation of the enzyme). It may thus be possible that Se may be more toxic for patients suffering from Se deficiency than for patients of more normal Se status.

### Health benefits of enhanced Se intake

Epidemiological studies from the USA have shown that mortality caused by cancer and cardiac diseases has been negatively correlated to the intake of Se, and the states in the USA with the lowest Se intake had the highest mortality of cancer and cardiac diseases (3,50–53). The US states with the highest Se intake in 1960 had the lowest mortality of cancer and cardiac diseases (3,50–53). These states had about three times higher average Se intake than in Norway 30 years ago, if a linear correlation between human blood Se concentrations and Se intake can be assumed both for USA and the countries in northern Europe (see data in refs 3,52,53). For hypertensive deaths in 1959–1961 among white males in the age group 55–64 years, average mortality from hypertension was only about half as high in the states with highest Se intake compared with the states with lowest Se intake (50), at a time when the average Se intake in the low-Se states in USA (judging from human blood Se concentrations) was at a similar level to that of Norway during the late 1970s (53) and for Finland today (54) – i.e. at a similar level as in the most Se-rich states in Europe. But there was much variation in hypertensive mortality among different low-Se states in USA (50), indicating that other aetiological factors also must be important. For all cardiovascular and renal diseases, mortality among white men 55–64 years old was about 25% higher and for white women 55–64 years old about 30% higher in the low-Se states than in the states with the highest Se intake (50).

A meta-analysis of Se and coronary heart disease has recently been published; it included 25 observational studies (14 cohort and 11 case control studies) and 6 randomized trials (55). The pooled relative risk in a comparison of the highest with the lowest Se concentration categories was 0.85 (95% CI: 0.74, 0.99) in cohort studies and 0.43 (0.29, 0.66) in case-control studies. In observational studies, a 50% increase in Se concentrations was associated with a 24% (7%, 38%) reduction in coronary heart disease risk. In randomized trials, the pooled relative risk in a comparison of supplements containing Se with placebo was 0.89 (0.68, 1.17). However, even if Se concentrations in this meta-analysis were found to be inversely associated with coronary heart disease risk in observational studies, the authors are very cautious, saying that because observational studies have provided misleading evidence for other antioxidants, the validity of this association is uncertain (55). It should be noted, however, that Se is more than an antioxidant nutrient.

There are many reports of the health benefits of Se from foods. There are regions of the world (in China) where severe Se deficiency has been identified, and where supplementation with Se has reduced deficiency diseases, notably the cardiomyopathy called Keshan disease (25,26,56). This is an endemic form of dilated cardiomyopathy leading to congestive heart failure that is attended by electrophysiological disturbances and cardiac muscle degeneration. It principally affects children and women of child-bearing age. In the majority of the European countries and other parts of China, classical symptoms associated with severe Se deficiency diseases are not commonly reported, but the daily Se intake is lower than the recommended Se intake (46). In these countries Se supplementation of the diet may possibly improve several different measures of health (3,4,12,47,53,56,57), such as reduction in cancer risk (53,58–60), better immune function (61–64), reduction of blood lipid oxidation (65,66), anti-ischaemic protection (67–80), reduction of morbidity and mortality from HIV disease (81–98), anti-ageing effects (99–102), improved fertility (3,56,103–105) and protection against allergic diseases and asthma (106–111).

### The world needs to improve cost-efficiency in the health sector

The selenium intake needed for saturating the antioxidant capacity of blood plasma (65,66) and for optimal protection against cardiovascular diseases and cancer, as indicated by epidemiological observations (3,50–53), is greater than the typical intakes of many people in the world. Two prospective clinical studies have also shown significant protective effects of Se supplementation at a ‘supranutritional’ level against colorectal cancer, prostate cancer, lung cancer and liver cancer (59,60). Few populations have intakes approaching this ‘supranutritional’ level, i.e. 200 μg/day or more (14). Accordingly, low Se status is likely to contribute to morbidity and mortality due to cancer and cardiovascular disease, and increasing Se intakes could be expected to reduce cancer rates as well as mortality from ischaemic heart disease (50,52,53,58,59). The health economic implications, if it is possible to reduce total age-adjusted deaths from cardiovascular diseases and cancer by more than 20%, as implied by the geographic epidemiological studies comparing states with high and low Se intakes in USA (50,53), would clearly be enormous. However, it should be remembered that the world needs vast improvements of cost-efficiency within the health sector if it is to be possible for the industrial countries simultaneously to tackle both the challenge of providing adequate medical care for their aging populations and financing the profound technological and economic restructuring that will be necessary to avert a global climatic catastrophe caused by anthropogenic pollution.

### Official recommendations for selenium intake

During the late 1970s and early 1980s, there was a so-called provisional RDA for Se recommending a Se intake for adults in the range 50–200 μg/day as safe and adequate (112). The average Se intake in Norway at that time was high enough to satisfy the American recommendations, about 70–80 μg/day (53), but the average Se intake in Finland fell well below the American recommendations, being only about 30 μg/day (42) during years with good cereal harvests in Finland, so that imports were low, and the situation in Sweden was – and is – not much better (113–119). Because of concern that low Se intake might be an important contributory cause of high cardiovascular mortality, it was decided in Finland to start fertilization with Se (54,120,121), so as to enhance the average Se intake up to the level that was considered necessary to saturate erythrocytes with glutathione peroxidase (P. Koivistoinen, personal communication). However, the Swedish health authorities did not want to do the same. And when a Nordic expert group tried to agree on a common Nordic recommendation for Se intake, it was decided not to follow the American provisional RDA, but instead lower the recommendation enough that the average Se intake in Sweden should not fall below the new Nordic recommendation.

This ‘adjusted’ Se recommendation has been used until recently by the Nordic countries, while the recommended daily intake (following international guidelines) is now 40 μg for adult females and 50 μg for men (122). The new recommendations are not based on Se intakes needed for saturation of intracellular glutathione peroxidase-1 (as was done in Finland during the mid-1980s), but on the Se intake needed for saturation of glutathione perox-idase-3 in blood plasma. This extracellular enzyme is saturated earlier (at a lower Se intake) than intracellular glutathione peroxidase-1.

### Awareness of the health benefits of selenium

The level of awareness of Se's health benefits appears to be low in Australia (123), and this is likely to be the case in most countries. Hence it may be a task for health authorities to ensure that the common diet contains adequate amounts of Se.

Several governments are aware of the health benefits of Se, and are working to ensure that their populations have an adequate intake. Supplementation programmes have been started in a number of countries (124), but government agencies have supported the use of Se in fertilizers on a national scale only in Finland and voluntarily in New Zealand.

On the other hand, some nutrition authorities have hesitated to take such steps, considering there to be insufficient scientific evidence that an increased Se intake will result in disease prevention (125). A recent workshop on Se supports this view (despite the evidence for Se's importance, presented above), and concludes that there is no evidence in Europe to suggest that there have been adverse effects associated with the decrease in Se intake (126). In Australia, proposed fortification programmes with iodine and folate have higher priority.

### Strategies to increase Se concentration in the human diet

Increased human Se intake may be achieved in several ways, and strategies include increased consumption of higher-Se foods through use of Se fertilizers, increased consumption of plants that naturally accumulate much Se, sprouting seeds in Se-enriched media, plant breeding for enhanced Se accumulation, plant production in the most Se-rich areas, supplementation of livestock, food fortification and supplementation of individuals. Each of these is discussed briefly below.

## Selenium in plants

### Increased Se concentration in plants

#### Se forms in plants

Plants convert Se mainly into selenomethionine (Se-met) and incorporate it into protein in place of methionine because the genetic code and tRNA do not discriminate between the two. Se-met is the major selenocompound in cereal grains, grassland legumes and soybeans, while Semethylselenocysteine (SeMCYS) is the major selenocompound in Se-enriched plants such as garlic, onions, broccoli, sprouts and wild leeks. The selenocompounds identified in plants have been summarized by Whanger (127,128) to be selenate, selenite, selenocysteine, Se-met, selenohomocysteine, SeMCYS, Se-met selenoxide, gamma-gluta-myl-Se-methylcysteine, selenocysteineselenic acid, Se-proponylselenocysteine selenoxide, Se-methyl-Se-met, selenocystathione, dimethyl diselenide, selenosinigrin, selenopeptide and selenowax.

Plants accumulate varying amounts of Se; some plants accumulate Se in direct relationship to the amount available from the soil, whereas others (Se accumulators) may accumulate Se in concentrations orders of magnitude above that in the majority of species. As much as 80% of the total Se in some accumulator plants is present as SeMCYS (47). The Se content and the chemical form of Se within plants may be altered by manipulation of plant genetics or by agricultural production conditions. Se-met in cereals is the major form of Se intake by humans, and its concentration increases with increasing soil Se (129).

#### Different Se forms in plants may affect biochemical pathways in man

It should be noted that Se-enriched plants contain mixtures of different Se components that are metabolized uniquely and can therefore affect multiple pathways, for example to inhibit carcinogenesis (46), and the chemical forms of Se in plants may partially dictate the metabolism of Se by the animal or human that consumes the plant. Therefore, attempts to maximize Se content in plants may have unintended consequences and must be carefully monitored (130).

## Selenium as a soil amendment in fertilizers

### Fortification of commercial fertilizers used in the food production chain was chosen to ensure an increased Se intake for the whole population in Finland

The Finnish supplementation programme (54,121,131) has provided experience with this approach to increase Se concentration in the diet, and it has been shown to be an effective and safe approach to raise Se levels in a human population. Fortification of Se in fertilizers in Finland was a well documented ‘study’ where a whole country has participated. In Finland daily Se intake in the 1970s was around 30 μg per day (42) following good summers (but much higher during years with crop failure because of poor weather conditions during the summer, so that much grain had to be imported). This was much lower than the recommended safe allowance at that time. It was also known that Se intake was different in different parts of Finland, with the lowest intakes being found in those parts of the country (especially in the east) where cardiovascular mortality was highest. As Finland's incidence of heart disease was the highest in the world, it was linked to findings from China, where the cardiomyopathy, Keshan disease, occurred only in Se-deficient areas, as well as with the experience both from Finland and other Nordic countries concerning severe diseases (including cardiomyopathy) caused by selenium and/or vitamin E deficiency in domestic animals.

Also, by the 1980s the studies that showed associations between low Se levels in soil/plants/ humans and higher incidence of cancer were published, giving further support to increasing the Se levels. Further, the Finnish authorities also wanted to avoid the risk that low Se in food could present a trade barrier for food exports. Selenium transfer to plants and Se transfer from feed to animal products were studied in Finland, and several methods were tested. In acid soil conditions Se from sodium selenate was more readily taken up by the plants than Se from sodium selenite. The Finnish authorities decided to add sodium selenate to multi-nutrient (NPK) fertilizers for all the field and horticultural crops at rates of 6 μg/kg fertilizer for grasses, and 16 μg/kg fertilizer for cereal and horticultural crops (54). After some adjustments of the dosage, it was decided to use 10 μg Se per kg fertilizer in 1998 (54). Agronomic approaches to Se enhancement were shown to be effective in Finland, and dietary Se intakes trebled and plasma Se concentrations nearly doubled within 3 years of the programme's commencement (131).

Globally, fertilization programmes similar to that in Finland may be conducted in low-Se areas. Plants grown in soil fortified with Se will be enriched in Se, and these plants may either be used directly for human consumption, or as animal feed. Thus both animals and all products in the food chain such as meat, milk and eggs will be enriched in Se.

Selenium supplementation to plants may also enhance the production and quality of edible plant products, by increasing antioxidant activity of the plants, as shown in tea leaves (132) and in rice (133), alter glucosinolate and sulphoraphane content (134), as well as increase plant growth (135) and fertility (136). Therefore Se fertilization may not only be beneficial for nutritive value in the food chain, but also for crop quality and, under certain circumstances, yield.

### Recovery of selenium added in the fertilizer

In the soil, Se is rapidly reduced to insoluble forms, and reports from Finland show that usually less than 10% of the applied Se was taken up by the crop. The Se not taken up by plants readily after application was apparently unavailable for crops growing later in the season or the next year (137). Others have found a better recovery of Se applied in fertilizers to grain, e.g. 18% recovery (33). A study in New Zealand found Se fertilizer to be most efficient if applied when the crop is beginning to grow rapidly in the spring (138).

## Selenium applied to plant leaves

Another method of transferring Se to plants is foliar application of Se, either as sodium selenate or sodium selenite. Foliar application was shown to be several times more efficient than application in fertilizers (54), but riskier as Se uptake by the crop depends on spraying conditions. Curtin et al. (138) also showed that foliar spray gave a high recovery. However, Lyons et al. (33) found foliar application to be less efficient than application to soil at planting (at application rates of 40 and 120 g Se/ha) in Australian trials.

The solutions commonly used in foliar application contain sodium selenate, which is highly toxic. Health and safety precautions must therefore be taken during its on-farm use.

## Seed treatment with selenium

Seed can be treated by applying sodium selenate directly on the seed surface. This method has been described by Gissel-Nielsen (139). However, it has not been shown to give efficient recovery of Se (138,139).

## To grow plants in selenium-rich soil

High soil Se concentration is not sufficient for high uptake in plants; bioavailability of Se in the soil must also be good. Therefore if areas with naturally Se-rich soils are to be exploited for cultivation of Se-rich food products, it is important to avoid cultivation methods (e.g. using Se-poor and P-rich commercial fertilizers) that may lead to impairment of the bioavailability of soil Se for uptake by the plant roots.

Growing plants in high-Se soil, or soil fertilized with Se, can produce Se-rich products that may be regarded as functional foods. These high-Se products may be ‘diluted’ with products grown on low-Se soil to obtain a desirable Se concentration in the marketable product, as is done with the Laucke flour, where high-Se wheat was grown by an Australian milling/food company, and blended with lower-Se wheat to provide flour, bread and ex-panded-grain biscuits containing 1.2–3.0 μg/kg Se in the products (141).

High premiums have been paid by European buyers of high-Se wheat grown in North Dakota (R. Welch, personal communication, 2004); however, beef produced on fields with supranutritional Se levels had unchanged production and carcass characteristics (142). Plant production in the most Se-rich areas is also practised in China, where an elixir is made from high-Se tea (14). Potatoes with increased Se concentration are produced in Australia, containing 70 μg Se per kg, thus a serving of 250 g potatoes would provide 17.5 μg Se, or about 25% of the recommended intake in Australia (the RDI for adult men is 70 μg and for women 60 μg Se/day) (143). Beer can be strategically biofortified with Se, as men are generally the highest beer consumers, and show the greatest benefits of increased Se intake. Biofortification of barley with Se has been found to be effective in producing high-Se beer (50–100 μg/l) (144).

## Plant species vary in Se accumulation

Plant species vary widely in Se uptake and accumulation. Primary Se accumulators such as *Astragalus bisulcatus* L. and *Stanleya pinnata* may contain as much as 10–15 g Se per kg DW (145,146). Studies have found a 15-fold variation in Se-accumulating ability among *Brassica* vegetables (14). Some mushrooms may accumulate Se and an Se-accumulating soybean cultivar has been identified (147). However, some of the forest mushrooms may also be very good accumulators of Se-antagonistic toxic metals, such as cadmium, which might be important – especially in forests severely affected by toxic metal deposition from acid rains, as in parts of Southern Norway (20), or when the bedrock is cadmium-rich Cambrian bituminous shale (alum shale), as in some districts in Norway and Sweden. The Se concentration varies somewhat within the plant, and in *A. bisulcatus*,Se was highest in the young leaves. It has been shown that Se hyperaccumulated in trichomes, present in the organic forms SeMCYS (53%) and gamma-glutamyl-MeSeCys (47%). In the young leaf itself, there was 30% inorganic Se (selenate and selenite) in addition to 70% SeMCYS. In young *S. pinnata* leaves, Se was highly concentrated near the leaf edge and surface in globular structures of epidermal cells, and both SeMCYS (88%) and selenocystathionine (12%) were revealed inside leaf edges. The high concentration of Se in the plant periphery may contribute to Se tolerance and may also serve as an elemental plant defence mechanism (146).

Se accumulator plants may be a good resource for Se. However, Se from broccoli may not be as bioavailable as Se from inorganic salts or Se-met for restoration of tissue Se concentration or selenoprotein activity in Se-deficient rats (148,149). Theoretically, it might be possible to use Se accumulator plants growing on naturally Se-rich soils for ‘biomining’ – with conversion of non-nutritional selenium compounds found in the Se accumulator plants into Se compounds more suitable as food ingredients after the plants have been harvested.

Exploiting the genetic variability in crop plants for micronutrient density may be an effective method to improve Se intake in human nutrition, and use of plants that naturally contain more Se than others, or breeding plant and crop varieties with enhanced Se-accumulation characteristics, may be plausible approaches to increase the Se concentration of the human diet. Several studies suggest that it is possible to breed cultivars with enhanced Se uptake and/or retention. The diploid wheat (*Aegilops tauschii* L) and rye were higher than other cereals in grain Se density, as shown in both field and hydroponic trials (150). However, when the topsoil is too severely depleted with regard to some nutrient element (e.g. as a consequence of accelerated soil erosion or too frequent anthropogenic fires), it is not possible to compensate for this by plant breeding or bioengineering to improve uptake kinetics in the plant roots – except by increasing the over-all development of the root system and making the roots go much deeper than before. The burglar cannot steal from a room that is empty.

If the problem of nutrient element deficiency in the topsoil has arisen as a consequence of too much disruption of natural systems of local biogeochemical recycling and retention, causing much more leakiness of the system than before (through enhanced soil erosion and leaching, or too much S and Se going up into smoke), the only viable long-term solution (especially for farmers so poor that they cannot afford to buy commercial fertilizers) is to try to re-establish agricultural ecosystems that are more similar to the natural ecosystems they replaced in the sense that the rate of leakage of nutrient elements is reduced to a minimum. This can be achieved using methods such as terracing (151,152), agroforestry, and reducing anthropogenic fires to a minimum (to minimize volatile losses of S, Se and N when plant material is burnt).

## Germination

### Biotransformation of inorganic selenium into organic selenium forms during germination

Nutritious products can be formed by enriching sprouts with inorganic forms of Se. As shown by Diowksz et al. (153), Bryszewska et al. (154) and Lintschinger et al. (155), inorganic Se added to grain that thereafter germinates will result in organic Se compounds in the sprouts. During the growth of the sprouts, Se is incorporated in the newly synthesized protein. Rye grain treated with selenite during germination has been shown to accumulate 55 μg/kg Se, which had all been converted to organic forms, mostly Se-met (154). The Se-rich rye (55 μg Se/kg) was then blended with ordinary rye to make bread with a final Se concentration of 4 μg/kg. This bread was eaten by volunteers in an intervention study and shown to be a good source of dietary Se (154).

Other applications of Se-rich germinated seeds may be considered, such as using bean sprouts, alfalfa-lucerne, etc., which can be mixed into various food products. Germination in wheat, alfalfa (*Medicago sativa*) and sunflower (*Helianthus annuus*) seeds has been reported (155), and uptake rates of inorganic Se have been studied. Conversion into organic forms showed that wheat and alfalfa were less resistant than sunflower and enriched Se up to concentrations of 100 and 150 μg of Se/kg of dry mass, respectively. The metabolism of the selenate was inversely related to the total uptake rates. It was also shown that sunflower sprouts were the most resistant and had the highest uptake rates (up to 900 μg/kg), but almost 100% of the Se was extracted with water and found to be non-metabo-lized selenate (155). Others have found that selenized alfalfa sprouts contain both Se-met and SeMCYS, and also an additional Se-containing species, a derivative of Se-2-propenyl selenocysteine, was possibly present (156).

An Se-containing peroxidase from barley germinating on an Se-containing artificial medium has been isolated, but the form of Se in the peptide was shown to be Se-met (157). The amino acid composition of this enzyme was similar to those peroxidases from other sources and both the Se-containing peroxidase from germinating barley and horseradish peroxidase had the same ESR signals as iron protoporphyrin, suggesting that the germinating barley Se-containing peroxidase is one of the peroxidase isoenzymes (157).

## Livestock

### Selenium supplementation

Selenium takes part in essential functions and is necessary for growth and survival for all animals. It is shown in general that the bioavailability of inorganic Se, mostly as selenite, is lower than the organic forms of Se such as Se-met (158). Absorbed inorganic forms (selenate, selenite) will in humans and animals undergo reductive metabolism yielding H_2_Se, which is the starting point for the production of selenoproteins such as glutathione peroxidase, selenoprotein P, selenoprotein W, thioredoxin reductase and various iodothyronine deiodinases (46). There is no known pathway in animals for synthesis of Se-met from inorganic Se (47), even though in ruminants it is possible for rumen microorganisms to convert inorganic S and Se into methionine and Se-met.

Se-met is a naturally occurring amino acid that represents the major nutritional source of Se for higher animals and humans. It can be incorporated into body proteins in place of methionine, making a reversible Se storage depot in organs and tissues. Enhancement of muscle protein catabolism in response to infectious disease must be expected to lead to mobilization not only of amino acids and zinc, but also Se from the skeletal muscles, which may help to stimulate immune functions (61–64) at the same time as it may also help to improve the tolerance of host cells against some of those reactive oxygen species and reactive nitrogen species that are used as weapons against the pathogenic invader. This property of functioning as a mobilizable storage form of Se is not shared by any other naturally occurring seleno-amino acid and Se-met may be regarded as a semi-essential nutrient (159).

The normal Se requirement has been set at 0.1 μg/kg DM feed, but the dietary requirement varies among animal species (160). The requirement for optimal protection against leg weakness (Bewe-gungsstörungssyndrom) in rapidly growing swine is reported to be about seven to eight times higher (161). The Finnish recommendations for Se for cattle are 0.1 μg/kg feed DM, for pigs 0.2, for poultry 0.2 μg/kg DM. The maximum permitted Se in compound feed or daily rations is 0.5 μg/kg DM in Finland (162).

Supplementation strategies to increase dietary Se intake by livestock, to secure animal health and prevent Se deficiency, and also to increase Se levels in meat, eggs and milk, include Se fertilization of pastures, dietary supplementation via feed concentrate rations, and direct administration (drenches, slow release reticulum/rumen ‘bullets’, injection).

### Addition of Se to commercial animal feed concentrates

Various chemical forms of Se have been examined as supplemental sources, with sodium selenite being the earliest and most used compound of choice. Supplementation of livestock with inorganic Se will secure the Se requirement for the animal itself, preventing it from Se deficiency disease.

In poor countries, where it is important to find practical methods of nutrient element distribution as cheap as possible, one could possibly use salt licking stones with Se added in the form of Se-met, and also such other mineral nutrients (e.g. iodine) that may be deficient in the area concerned.

Because of concerns expressed by the European Community Scientific Committee on Food that Se-yeast supplements are poorly characterized, the addition of organic Se forms (as selenised yeast) to feed concentrate for domestic animals such as broilers, egg-laying hens, dairy cows, etc., is currently not permitted in EC countries (and at present the organic Se sources are not accepted as feed additives (162)). However, the organic Se product (Se-yeast) has recently been evaluated, and it has been approved by the Standing Committee, which will soon be published in the Official Journal.

## Supplementation of livestock (to increase selenium concentration in animal products)

### Se-enriched animal products

The production of Se-enriched animal products is increasing. The use of Se-yeast (Se-rich yeast containing high amounts of Se-met) to supplement the diet of cows has been shown to be considerably more effective than inorganic Se in raising the Se concentration in milk and cheese (163,164). It has been shown that egg Se content could be easily increased up to about 30–35 μg when organic Se is included in the hen feed at a level of 0.4–0.8 μg/kg feed (165), and clinical studies on humans consuming two Se-enriched eggs per day for 8 weeks showed a significant increase in Se level in plasma (165). By adding around 0.8 μg Se (organic forms) per kilo broiler feed concentrate, the Se concentration in broiler muscle may reach levels as high (own results) as those found in fish (cod contains about 0.4 μg/kg fresh weight, USDA database).

Meat quality may also be enhanced following organic forms of Se supplementation to feed, as drip loss of breast meat was found to be reduced in broilers fed organic Se in the diet as compared with selenite (166).

The consumption of animal products varies greatly between countries, and among cultures and socioeconomic classes. To ensure adequate Se intake in a population, enrichment of several commonly used foods and not only animal products would have to be done.

The annual worldwide consumption of meat is only about one-tenth of the consumption of cereals (Table I), or around 265 105 000 tonnes per year (167), thus Se supplementation of animals to increase Se concentration in meat would not be the most efficient way to increase it in the average person's diet. However, in most diets, the dominant food sources of Se are cereals, meat and fish. For some individuals meat and animal products are a major part of the diet.

**Table I:** World's total production of cereals, meat, fruit and vegetables, roots and tubers, pulses, oilseeds and nuts and fisheries (167).

Source	Tonnes	*Estimated Se (μg/kg)	‘Estimated mean’	Total Se production (tonnes)
Agriculture				
Cereals	2 227 980 000	0.05–0.6	0.1	223
Meat	265 105 000	0.05–0.30	0.1	27
Fruit and vegetables	1 392 253 000	0.002–0.08	0.05	70
Roots and tubers	711 682 000	0.002–0.08	0.05	35
Pulses	61 706 000	0.002–0.08	0.05	3
Oilseeds and nuts	146 353 000	0.002–19.0	0.04	5
			Total from agriculture	363
Fisheries				
Pelagic	40 664 000	0.3	0.3	12
Freshwater	36 147 000	0.3	0.3	11
Demersal	22 324 000	0.3	0.3	7
Molluscs	16 786 000	0.4	0.4	7
			Total from fisheries	37

* Estimated selenium (Se) concentration in food products from Combs (14). An ‘estimated mean’ is suggested by the authors.

For patients suffering from protein-catabolic conditions such as HIV disease and tuberculosis in poor countries, it is very important to improve the supply of cheap high-quality protein either from local agriculture or through improvement of the utilization of cheap fish resources for human consumption (which may be possible especially when using pelagic fish living in regions of oceanic upwelling, e.g. off the coasts of Namibia and Angola). Making Se-enriched animal products such as goat milk (from high-yielding dairy goats, e.g. by using salt licking stones containing Se-met) would appear a good strategy for improving the nutrition status of HIV patients in rural areas in Sub-Saharan Africa. It might help to correct several different nutrient deficiency conditions, being especially important in this context at the same time as it can be done cheaply in an ecologically acceptable way, especially when combined with agroforestry (and in hilly terrain also terrace construction) as methods of improving soil fertility.

### Direct administration of Se to livestock

Direct administration of Se supplements to livestock can be achieved with drenches, injections and slow-release rumen ‘bullets’, with inorganic selenite as the main Se form, often as barium salts. High pressure pelleted ruminal boluses with greater Se particle size aggregated into hollow pellets have been shown to have prolonged effects (168). The apparent absorption of Se that has been added to feed concentrate has been shown to be higher from concentrate than from hay (169).

### Administration of Se to animals via Se-enriched pasture

In Finland addition of fertilizers to pasture (6 μg sodium selenate per kg fertilizer) has increased the overall Se concentration in feed materials from μg Se/kg DM to 0.2 μg Se/kg DM feed (54). This meets the dietary Se requirement for the animal, and the Se concentration in animal products increases as well. The Se concentration in beef increased from less than 0.2 μg/kg DM in 1983, up to 0.6 μg/kg in 1989 and then down to about μg/kg DM, due to different doses of Se fertilization over time. The Se concentration in milk increased from about 0.05 μg/kg DM in 1983 to about 0.2 μg/kg DM in 2003 (170).

In New Zealand, sodium selenate is commonly applied as a prill to pastures (25). Slow release barium selenate has been applied to pasture at 10 g/ha, and this application form prevented subclinical Se deficiency in sheep for 4 years, whereas a single application of sodium selenate at the same concentration was effective for only 15 months (171). This application form therefore may be a more resource-saving method than traditional Se fertilization of pasture.

## Direct selenium supplementation of food

### Addition of different selenium forms and doses directly to food (e.g. bread, flour, salt)

Direct application of Se to food is a resource-saving way to supplement with Se. In principle, both inorganic and organic Se forms might be used as food supplements, and the different forms in Se supplement pills have been tested in several intervention studies, and used by many individuals for several years without any apparent associated problems.

Staple foods such as cereal flours might be supplemented directly with inorganic or organic Se forms during the milling process. However, most countries have directives for adding nutrient supplements to foods, and advantages and potential risks of different Se forms and doses need to be assessed before implementation.

Selenium can be added to table salt to increase the Se intake in a population. In the Qidong region of China, more than 20 000 persons were given table salt fortified with 15 μg Se as sodium selenite per gram of salt. The salt provided about 30–50 μg Se/day. The supplementation lasted for 8 years and it resulted in a 35% reduction in the incidence of primary liver cancer (59).

If Se-met is to be added directly to food, precautions must be taken to avoid oxidation. Oxidized Se-met is less available for humans, as shown from the plasma Se concentration of participants in a clinical trial (172). They showed that Se-met in solution that was added to wholegrain wheat which was then heated to around 200°C to produce expanded-grain wafers was entirely oxidized to methionine selenoxide. Methionine selenoxide was found to be poorly absorbed from the gut of healthy volunteers, compared with Se provided from biofortified wheat, produced by foliar application of selenate. Selenium in the biofortified wheat was also mostly Se-met, but it was not oxidized by the heat treatment (172). Further studies are needed to investigate methods to prevent oxidation during processing, for example, by adding antioxidants or processing under inert gas, or heating at lower temperatures. It has been shown that Se-met selenoxide may be easily reduced back to Se-met by glutathione (173). Adding glutathione or combining the products with foods rich in glutathione may be an option to prevent oxidation. If extreme heat is required, and inorganic Se forms are disallowed, biofortification may be the only feasible option.

### Toxicity

As inorganic Se has no taste, smell or colour, there is always a risk of overdosing the nutrient when adding it to food products. The mixing procedures must therefore be under strict quality control to avoid this possibility. Mixing the Se compound used with some non-toxic (biologically inert) pigment (e.g. Prussian blue) might also be used as a precautionary measure.

Products that are high in Se and that are produced either in seleniferous areas, under high Se fertilization of soil or leaves, or germinated seeds containing high Se concentrations, to be later blended with ordinary products, will not have a special smell or taste. Rigorous control routines must be adopted to prevent these products being consumed before dilution.

## Selenium pills

Consumption of Se pills is an option to increase the intake of Se in individuals. Selenium supplements are available as sodium selenite, sodium hydrogen selenite, sodium selenate, Se-enriched yeast and Se-met (46). Many individuals wish to increase their Se intake, but the problem with supplementation of nutrients via pills to the whole population is that many people – and often those who would gain most benefit (e.g. male heavy smokers) – are unlikely to take the supplements. Therefore, the best way to prevent dietary shortfalls of the nutrient may be to have adequate amounts of Se in commonly consumed food products (even though pills may still be used for therapeutic purposes in situations where especially high intakes might be useful, e.g. when selenium is used as an antidote).

## Global Se budget

The annual production of agricultural products, fish and seafood in the world are shown in Table I (167). Table I also gives an estimation of natural variation of Se concentration in different food products (14). By multiplying our estimated Se concentration by the annual amount of the different food products produced, the world's annual Se production from agriculture and fisheries is found.

Worldwide, cereals are the most important dietary source of Se, followed by fruit/vegetables and roots/ tubers. Total Se intake from cereals is about 10 times higher than from meat (Table I).

Table II shows the world's resources of arable land, permanent crop land and pasture, along with amounts of fertilizer produced and consumed annually.

**Table II:** World's total resources of arable land, permanent crops and pasture; world's fertilizer production and consumption, and numbers of cattle, buffaloes, sheep and goats (167).

Resources	Amount
Arable land	1 402 317 000 ha
Permanent crops	138 255 000 ha
Pasture	3 432 834 000 ha
Fertilizer production	155 057 000 tonnes
Fertilizer consumption	147 917 000 tonnes
Cattle and buffaloes	1 529 110 000 head
Sheep and goats	1 888 736 000 head

An Se budget can be made, by summing the world's production of Se from mining and agricultural products and fisheries, as shown in Table III. An estimate of how much Se is needed annually to cover the Se requirements for the world's human and livestock population is calculated by multiplying world population with the recommended Se intake (three different levels are suggested in the table), using both the recommended amount of about 50 μg Se per day, 100 μg per day, and the daily amount that may be recommended to prevent certain types of cancer, 250 μg Se per day (Table III).

**Table III:** Estimated annual selenium (Se) budget; tonnes Se produced from mining industries (2) and Se produced in agriculture and fisheries.*

Annual Se budget	Tonnes produced	Tonnes consumed
Se production		
Mining industries	2300	
Cereals, meat, vegetables, fruit, fish	400	
Se need/consumption		
Human requirement, prevention of Se deficiency (0.05 μg/day/person)		119
Domestic animal requirement (3 500 000 000 head–365 – 0.100 μg)		130
Total	2700	249
If human requirement is higher (and animals unchanged):		
Prevention of several diseases (0.1 mg/day/person)		367
Prevention of certain cancers (0.25 mg/day/person)		723
These requirements may be met in several ways:		
Fertilizing one-third of arable land (20 g Se/ha)		1000
Fertilizing one-third of pasture (20 g Se/ha)		2000
Direct supplementation of one-third of all humans (0.05 mg/day)		40
Direct supplementation of one-third of all animals (0.1 mg/day)		40
Supplementation of one-third of all animals via feed (0.1 mg Se per kg feed 1 100 000 000 5 kg)		550

* An estimate of tonnes Se needed/consumed by the world's population of humans (6.5 billion people) and livestock is also shown. Examples of how to meet the world's Se requirement are shown.

Table III shows that the estimated world's annual Se production via agriculture and fisheries is about 400 tonnes. This amount of Se – if evenly distributed – is enough to supply 100 μg per day for each person and each head of livestock in the world, but it is not enough to supply the higher rate that may protect against cancer. As there are no reliable records of pigs and poultry numbers, their requirements are not included in this discussion. As the intake is unevenly distributed throughout the world, the requirements would be actually higher than these estimates. In respect of uneven distribution Combs (14) has estimated the need for supplementation to approximately 500–1000 million people to prevent Se deficiency, not to mention the vast majority of people that are considered to be subclinically deficient (14).

As the actual requirement for optimal prevention of cancer and other diseases is claimed to be higher than 0.05 μg/day (around 0.2 μg/day or more), the annual Se production via agriculture and fisheries may be too small to cover the human requirement, and supplementation of Se would be likely to be needed in many areas of the world.

## Selenium budgets in various scenarios

### Fertilizing arable land

Selenium fertilizer programmes for the Se-poor fraction of the world's productive area would be an effective way to increase Se content in human food and animal diets (and may also benefit plant quality and growth). However, the amount of Se that would be needed globally each year would be large, for example, if applying about 20 g Se/ha to one-third of the productive arable land area, it would annually amount to about 1000 tonnes Se, which is more than a third of the current annual Se production.

### Fertilizing pasture

World pasture resources are 3 432 834 000 ha, or more than double the arable land area. If a third of the pasture also were to be fertilized with the same amount of Se per ha, it would draw on another 2000 tonnes, or about two-thirds, which would require an annual production in excess of 4000 tonnes.

As the world's production of meat is low and a large part of the world's inhabitants consume very little meat, this method of supplementation would benefit only a relatively small proportion of global population.

### Add selenium to the current compound fertilizers

The annual production of fertilizers is around 155 057 000 tonnes. If Se is added to fertilizers at 10 μg per kg fertilizer, about 1500 tonnes would be needed annually, which is about half of the world's Se production.

### Supplementation of animals via feed concentrate, supplement mixtures or boluses

Selenium may also be supplied directly to the animal either via feed concentrate or via oral supplementation methods (as tablets, supplement mixtures or boluses) if the pasture is Se-deficient.

The dietary requirement of Se varies between animal species, and according to age and pregnancy (160). Using an average requirement of 0.1 μg/kg feed DM, and an average feed intake for small and large domestic animals of 2 kg DM per day, about 0.2 μg Se is needed per animal per day, or about 0.1 g per year. If about 1 billion animals are supplemented, about 100 tonnes would be needed or about 4% of the annual Se production.

A somewhat higher figure is required to give supplementation via boluses: bolus for cattle contains 0.3% selenite, and for sheep and goats 0.15%. The bolus size is from 50 to 200 g, and about four boluses are needed per year. Thus an average of about 0.5g Se is given per animal per year. With the number of cattle, buffalo, sheep and goats being about 3 300 000 000 head, for a third of the animals to be supplemented (about 1 000 000 000), this amounts to 500 tonnes, or about 20% of the annual Se production.

With a supplementation programme of domestic animals, part of the Se will be lost through faeces. The manure may be used as fertilizers and thus part of the Se here can be incorporated in plant material.

### Increase selenium in food by blending in selenium-rich sprouts

Selenium has been shown to be taken up by seeds during germination. The efficiency of Se uptake by the seed during the germination process has been shown to be high. Processing may be optimized in such a way that very little Se is lost during the germination process, and theoretically all added Se might be taken up by the seed. This method of Se supplementation (late in the food chain) may turn out to be far more effective than any other way of biofortification of plant products. Assuming that about a third of the world's population of 6.5 billion would benefit from having a Se supplement daily of 100 μg Se (or 0.036 g per year), 72 tonnes would be needed. If some of the food is discarded, an estimate of about 100 tonnes would be needed. This is only about 4% of the world's annual production of Se, and this way of biofortification is therefore much more efficient than via traditional agriculture. Further studies and research on the different forms of Se that may be formed during germination and their effects in the organism are needed.

### Increase selenium content in food by adding inorganic or organic selenium forms directly in the food

Food industries may enrich various types of food with inorganic Se or with organic Se forms. If Se-met is used, the potential problem of oxidation of Se-met must be avoided. As a third of the world's population of 6.5 billion would benefit from having a daily Se supplement of 100 μg Se (or 0.036 g per year), 72 tonnes would be needed. As mentioned above, some food will be wasted, and an estimate of about 100 tonnes would be needed (about 4% of the world's annual production of Se).

### Via supplementation by selenium tablets

If a third of the world's population of 6.5 billion took an Se tablet daily containing 100 μg Se (or 0.036 g per year), 72 tonnes would be needed or about 3% of the total annual Se production.

## Recommendation

By far, the most resource-efficient way to increase the Se intake in the world's population appears to be by adding Se to food products well along the production chain. An efficient and resource-saving method to biofortify plants appears to be addition of Se to seeds during germination to produce high-Se sprouts. These sprouts may then be added to various food products in concentrations so as to make new products biofortified with Se in optimal concentrations. The method of using Se-enriched germinated seeds in different food products looks promising. However, further research on the metabolic effects in the body of the different Se components that may be produced during germination is needed.

Selenium fortification of commonly used food products such as cereals or table salt by the food industry is also resource-saving. Both inorganic and organic forms of Se may be used in fortification programs, except where proscribed by governments.

Selenium tablets for daily intake by individuals are another possible approach to increase Se intake in a population. This method carries a high risk of not reaching the whole population.

Selenium has to be supplemented to animals in some areas of the world to prevent Se deficiency diseases of the livestock. However, today mostly inorganic Se forms are given, that will not be converted to Se-met and stored in the muscle. Giving organic Se forms would give higher levels of Se-met in the products, and thus benefit the consumer.

Selenium added in commercial fertilizers is not efficiently taken up by plants, and the amount that is not taken up cannot be reutilized. This Se is therefore lost for the future. Large-scale fertilization programmes with Se-enriched fertilizers may be found to be wasteful and unsustainable. The Se resource must be managed for future generations. However, this method is documented to be successful in Finland. When increasing Se concentration in plants, the nutritive value of the entire food chain is likely to be improved. National plans on how to utilize Se resources efficiently in each country would be desirable.

At the same time, it may be important to try to minimize industrial consumption of Se for all such purposes where it is not strictly needed. Total production of Se can be increased by exploiting the Se content of sulphide ores of metals other than copper and also the Se content of coal. SO_2_ emissions from plants producing metals such as nickel from sulphide ores have been considered mainly as a serious local pollution problem, killing forest vegetation and potentially fish in the vicinity of the smelter. But it also represents an enormous waste of a nonrenewable resource that should be considered vitally important for the health of future generations. Efforts should therefore be taken to extract the Se content of these ores and not sell them too cheaply for industrial use. Instead it would be better to stockpile much of it in well-guarded underground stores for the benefit of generations to come.

Possibilities of extracting Se (and perhaps other rare elements as well) as a by-product during purification of effluent gases from coal combustion should also be considered. Great progress has been made over the last 25 years towards reduction of industrial SO_2_ emissions in Europe and North America. But the main objective has been to try to eliminate a serious pollution problem, which was killing freshwater fish and damaging forests. Consideration was not given to the importance of Se and S as valuable mineral nutrient resources, indispensable for the life and optimal health of human and animal populations.

In the future, it will also be necessary – to avoid catastrophic global warming – to radically reduce CO_2_ emissions from combustion of oil, gas and coal. This is a great technological challenge – how to find good integrated solutions that may permit construction of coal-based power plants that are pollution-free (because CO_2_ is stored underground in suitable reservoir rocks, e.g. in the North Sea), while also permitting optimal recovery of Se and other valuable minor components that are commonly highly enriched in coals.

## Conclusions

The world's rare Se resources need to be managed carefully so that this vulnerable resource is not squandered. Selenium is essential to humans, for animal health and animal product quality, and may also improve plant growth and quality. Large bio-fortification programmes where Se is added in commercial fertilizers may be a method that is too wasteful to be applied to a large part of our planet, as much of the Se used thereby will be lost for future utilization. Biofortification of plants and animal products needs to be considered carefully, and care must be taken to avoid waste. These supplementation methods should be evaluated by each country in the light of Se resource planning and sustainability.

Direct addition of Se compounds to food (process fortification) can be undertaken by the food industry. If Se-met is added directly to food, oxidation due to heat processing has to be avoided. New ways to biofortify food products by adding Se-enriched sprouts, produced through germination of seeds in Se-rich media, is an interesting concept. Special considerations are needed for supplementing the diets of populations in developing countries, where access to processed and fortified foods is limited.
